# Enhancing structured data generation with GPT-4o evaluating prompt efficiency across prompt styles

**DOI:** 10.3389/frai.2025.1558938

**Published:** 2025-03-26

**Authors:** Ashraf Elnashar, Jules White, Douglas C. Schmidt

**Affiliations:** ^1^Department of Computer Science, Vanderbilt University, Nashville, TN, United States; ^2^Department of Computer Science, William & Mary, Williamsburg, VA, United States

**Keywords:** structured data generation, prompt engineering, GPT-4o, JSON, YAML, Hybrid CSV/Prefix, token efficiency, cost-effective AI

## Abstract

Large language models (LLMs), such as GPT-4o, provide versatile techniques for generating and formatting structured data. However, prompt style plays a critical role in determining the accuracy, efficiency, and token cost of the generated outputs. This paper explores the effectiveness of three specific prompt styles–JSON, YAML, and Hybrid CSV/Prefix–for structured data generation across diverse applications. We focus on scenarios such as personal stories, receipts, and medical records, using randomized datasets to evaluate each prompt style's impact. Our analysis examines these prompt styles across three key metrics: accuracy in preserving data attributes, token cost associated with output generation, and processing time required for completion. By incorporating structured validation and comparative analysis, we ensure precise evaluation of each prompt style's performance. Results are visualized through metrics-based comparisons, such as Prompt Style vs. Accuracy, Prompt Style vs. Token Cost, and Prompt Style vs. Processing Time. Our findings reveal trade-offs between prompt style complexity and performance, with JSON providing high accuracy for complex data, YAML offering a balance between readability and efficiency, and Hybrid CSV/Prefix excelling in token and time efficiency for flat data structures. This paper explores the pros and cons of applying the GPT-4o LLM to generate structured data. It also provides practical recommendations for selecting prompt styles tailored to specific requirements, such as data integrity, cost-effectiveness, and real-time processing needs. Our findings contribute to research on how prompt engineering can optimize structured data generation for AI-driven applications, as well as documenting limitations that motivate future work needed to improve LLMs for complex tasks.

## 1 Introduction

### 1.1 Enhancing structured data generation with GPT-4o

Accurate and efficient generation of structured data from unstructured text (Moundas et al., 2024) is essential across domains like business intelligence, data analytics, and automation. Large language models (LLMs), such as GPT-4o, help automate this process, enabling the creation of structured outputs (Dagdelen et al., 2024) in prompt styles like JSON, YAML, and Hybrid CSV/Prefix. These styles ensure compatibility with software systems and data pipelines, but the effectiveness of structured data generation depends largely on prompt engineering decisions (Liu et al., 2023). The choice of prompt style can significantly affect the quality of the output, as well as the associated token costs and processing time.

### 1.2 Overview of prompting styles for structured data generation

Prompt styles play a critical role in determining the efficiency and accuracy of structured data generation. In this study, we compare three widely used prompt styles:

- **JSON** (JavaScript Object Notation) is a hierarchical data format that ensures strict adherence to structure. It is particularly useful for applications requiring nested attributes and structured data consistency. JSON can be verbose, however, leading to increased token consumption and higher processing times.
- **YAML** (Yet Another Markup Language) is a more human-readable alternative to JSON while preserving structure. It eliminates the need for excessive syntax (such as curly braces and quotation marks), making it more compact. While YAML improves readability, it may introduce minor inconsistencies in formatting that impact accuracy in structured data extraction.
- **Hybrid CSV/Prefix** is a format that combines CSV (Comma-Separated Values) with prefixed identifiers, ensuring compact representation while maintaining structural integrity. It is particularly efficient in terms of token usage and processing time, making it ideal for tabular or transactional data. However, it lacks hierarchical representation, limiting its suitability for complex structured datasets.

Despite GPT-4o's potential for structured data generation (Tipirneni et al., 2024), little is known on how to optimize prompt styles for generating high-quality outputs. JSON and YAML are widely used due to their structured nature and readability, but they can be verbose and resource-intensive, especially for large, complex datasets. Hybrid CSV/Prefix is a more concise alternative, striking a balance between simplicity and structure, making it suitable for flat data representations. However, selecting the right prompt style to optimize accuracy, efficiency, and cost remains a challenge.

### 1.3 Our approach → Analyzing the effectiveness of prompt styles with GPT-4o

This paper investigates the performance of three prompt styles–JSON, YAML, and Hybrid CSV/Prefix–used with GPT-4o for structured data generation. Our study evaluates these prompt styles across three scenarios: personal stories, receipts, and medical records. Each prompt style is assessed based on three metrics: accuracy (how well the generated outputs match expected attributes), token cost (an indicator of computational resource consumption), and processing time (efficiency in generating results). By focusing on GPT-4o, our study provides a detailed analysis of its strengths and limitations in handling diverse structured data tasks.

This paper makes three contributions to prompt design for structured data generation using GPT-4o:

- We designed an experiment that generates randomized datasets in three distinct scenarios (personal stories, medical records, and receipts) to evaluate how effectively each prompt style captures the required structure across diverse and context-specific scenarios. Datasets for personal stories contain attributes representing individual characteristics and paired them with corresponding valid narratives. Datasets for medical records contain medical attributes to produce valid and realistic entries. Datasets for receipts contain attributes to create valid examples reflecting real-world purchases.
- We used these three datasets to compare the outputs generated by GPT-4o with expected results. Accuracy was measured based on strict adherence to the original data attributes and values, ensuring the generated structured data matched the intended JSON, YAML, and Hybrid CSV/Prefix prompt styles.
- We analyzed the results by measuring the token usage and time needed for each prompt style. The findings are presented via graphs that visualize key metrics: accuracy, token cost, and time efficiency. These visualizations–specifically the *Technique vs. Accuracy, Technique vs. Token Cost*, and *Technique vs. Time* graphs–highlight the pros and cons of each prompt style in generating structured data.

### 1.4 Paper organization

The remainder of this paper is organized as follows: Section 2 summarizes the open research questions addressed in our study and outlines its technical approach; Section 3 explains our experiment design, datasets, and testbed environment; Section 4 analyzes the results of experiments that evaluate the efficiency, accuracy, and cost-effectiveness of different prompting strategies for structured data generation using GPT-4o; Section 5 provides a comparative analysis of the performance of GPT-4o on our datasets; Section 6 compares our research with related work; and Section 7 presents lessons learned from our study and outlines future work.

## 2 Summary of research questions

Our study formulated four research questions aimed at assessing the performance of different prompt styles for structured data generation using GPT-4o. Each question addresses a key aspect of prompt performance, with a focus on accuracy, efficiency, and cost-effectiveness. Given the increasing role of LLMs in structured data processing, our research aims to push the boundaries of prompt engineering by systematically analyzing how different styles impact structured data generation. Additionally, we investigate the broader implications of prompt selection on automation, scalability, and real-time processing constraints.

### 2.1 Q1: How do different prompt styles impact the accuracy and reliability of structured data generation?

This question evaluates the accuracy of each prompt style by analyzing GPT-4o's ability to generate outputs that faithfully represent the expected attributes and values in the test datasets. Accuracy is assessed not only based on strict adherence to predefined attributes but also on the robustness of structured outputs under varying dataset complexities and contexts. This allows us to explore whether certain prompt styles provide greater resilience to inconsistencies in real-world applications.

### 2.2 Q2: What is the token cost and computational efficiency associated with each prompt style, and how does it scale for large datasets?

Since token usage directly impacts GPT-4o's computational costs, this question extends beyond individual token efficiency to consider scalability. By tracking token consumption under varying dataset sizes, we assess whether different prompt styles exhibit consistent cost trends as data complexity increases. This insight is particularly relevant for AI-driven data extraction pipelines where computational overhead directly affects deployment feasibility.

### 2.3 Q3: How does each prompt style influence structured data generation speed and latency, particularly in real-time and batch-processing scenarios?

This question examines GPT-4o's response time under different prompt styles, focusing on both real-time applications (e.g., chat-based AI systems) and large-scale batch processing. Understanding the latency differences between JSON, YAML, and Hybrid CSV/Prefix prompts helps inform which prompt structures are best suited for high-throughput applications where minimal processing delay is required.

### 2.4 Q4: How well do different prompt styles generalize across multiple structured data contexts, and what are the trade-offs for specific use cases?

Given the diversity of structured data applications, this question investigates the adaptability of prompt styles across distinct data types, including personal stories, receipts, and medical records. Instead of solely evaluating static performance metrics, we analyze how well each style generalizes to new data configurations, providing insights into their broader applicability and limitations. This analysis ensures that our findings extend beyond controlled datasets to more practical, real-world applications.

By addressing these questions, our study contributes to the evolving understanding of prompt engineering for structured data generation, offering insights into how LLMs like GPT-4o can be leveraged to enhance structured data workflows in AI-driven environments.

## 3 Experiment design

To address the research questions described in Section 2, we designed a study involving randomized data generation, prompt formulation (Guo, 2023), interactions with GPT-4o (Wang et al., 2023), and validation of the generated outputs. This study evaluated the efficiency, accuracy, and cost-effectiveness of three prompting styles–JSON, YAML, and a hybrid CSV/prefix format–for generating structured data across three distinct contexts: personal stories, receipts, and medical records. To ensure a focused and detailed analysis, the study was conducted exclusively using GPT-4o.

**Figure 1:**
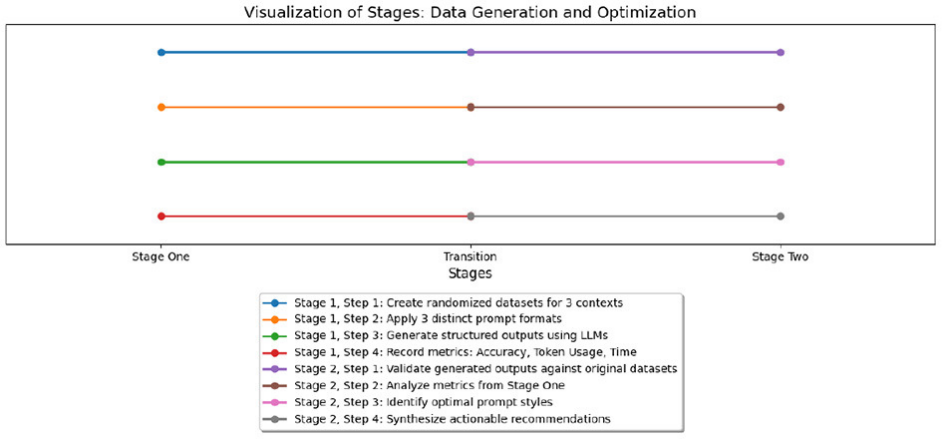
Visualization of study stages.

Systematically assessing the performance of each prompt style with GPT-4o identified the optimal style for structured data generation based on token usage, processing time, and accuracy metrics. This experiment was organized into two stages, as shown in Figure 1 and described below:

- **Stage One: the 3** **×** **3** **×** **3 framework for data generation and prompt testing**. This stage utilized a 3 × 3 × 3 framework with three specific contexts (personal stories, receipts, and medical records), three prompt styles (JSON, YAML, and Hybrid CSV/Prefix), and three key metrics (accuracy, token usage, and generation time). Randomized datasets were created for each context and the three prompt styles were applied to guide GPT-4o in generating structured outputs. Metrics were recorded for each combination of context, prompt style, and metric to evaluation performance accurately.
- **Stage Two: assessment and refinement**. This stage validated the outputs generated by GPT-4o against the original datasets to measure accuracy. The metrics collected during Stage One were analyzed to identify the most efficient and effective prompt styles for each data context. The findings were synthesized into actionable recommendations, emphasizing the trade-offs and advantages of JSON, YAML, and Hybrid CSV/Prefix prompt styles for structured data generation.

### 3.1 Stage One: the 3 × 3 × 3 framework for data generation and prompt testing

Stage One of our study applied a 3 × 3 × 3 framework to evaluate structured data generation systematically using GPT-4o. The goal was to create and validate diverse datasets (Fan et al., 2018) that simulate realistic data generation scenarios across these contexts, using the three prompt styles as instructions for GPT-4o. These datasets serve as the foundation for evaluating the effectiveness of each prompt style in Stage Two, as discussed in Section 3.2.

#### 3.1.1 Dataset overview and methodology

The dataset used for our experiment consisted of three data types: Personal Stories, Receipts, and Medical Records. Each data type contains 600 entries, leading to a total of 1,800 records. The dataset is structured using three different prompting styles: JSON, YAML, and Hybrid CSV/Prefix. To analyze the characteristics of the generated dataset, we compared the lengths of expected versus generated outputs across different prompt styles.

**Figure 2:**
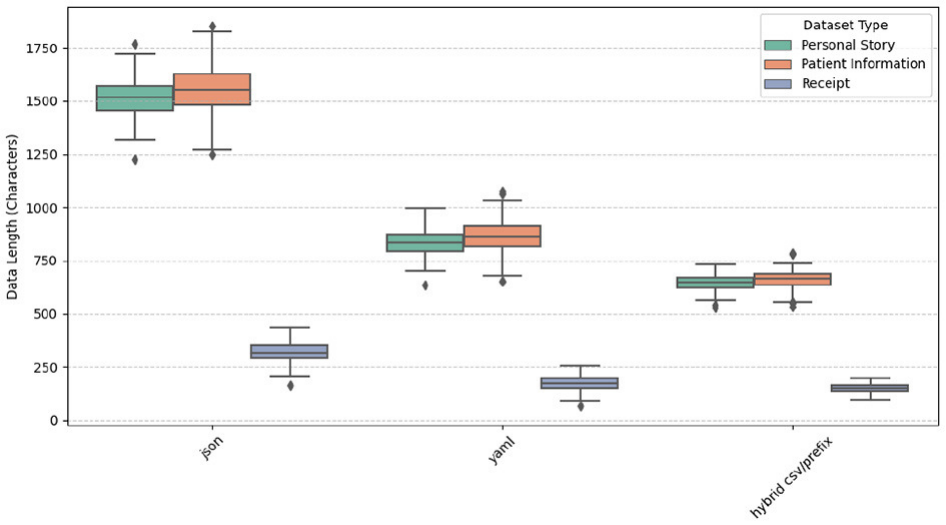
Variation in data size for JSON, YAML, and Hybrid CSV/Prefix.

**Figure 3:**
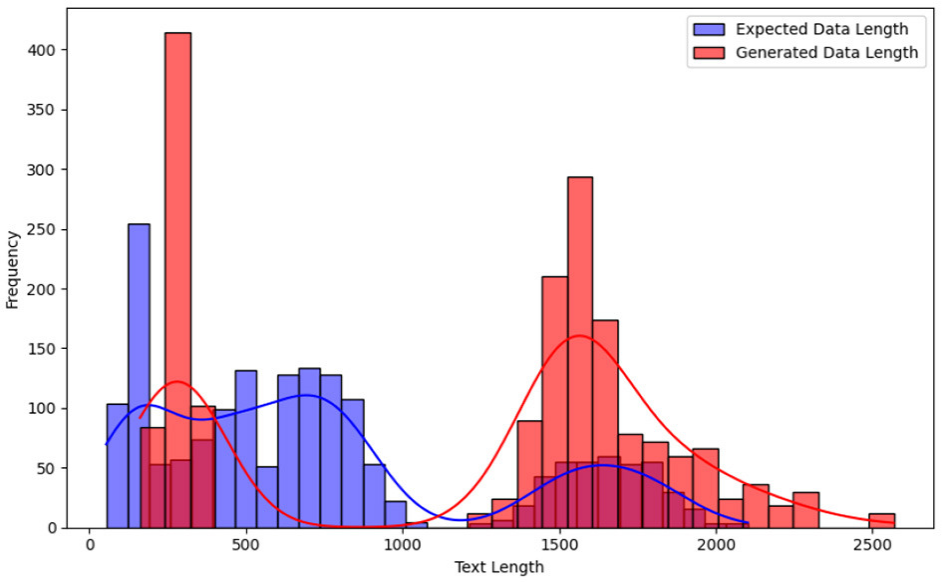
Expected vs. generated data lengths.

Figure 2 presents the distribution of output lengths across these prompt styles, highlighting key variations in structure and verbosity. Our analysis revealed that, on average, generated outputs were 462 characters longer than expected, with some cases extending up to 2,051 additional characters. Figure 3 presents a histogram with KDE (Kernel Density Estimation) comparing expected versus generated data lengths. This analysis underscores differences in verbosity and formatting across prompt styles.

**Figure 4:**
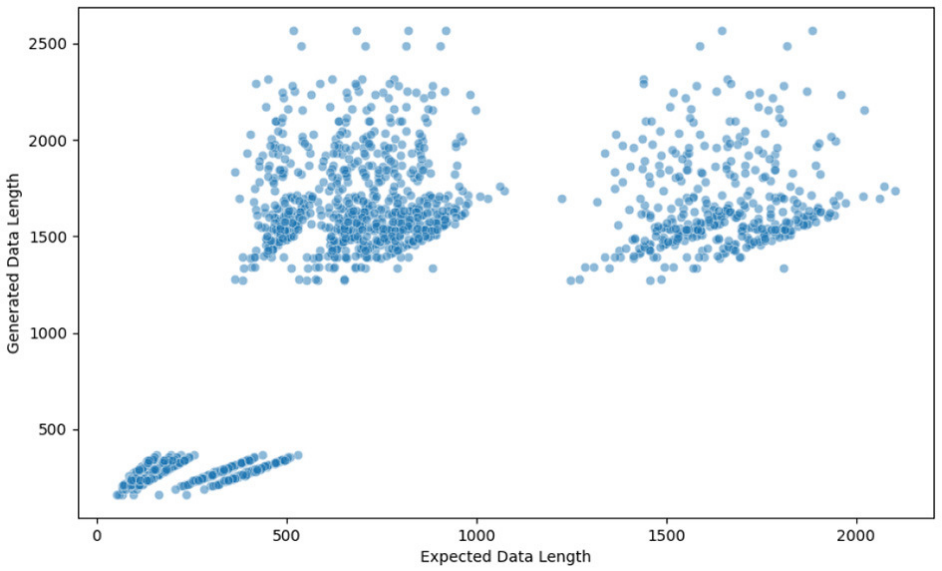
Distribution of data extraction prompt styles.

Figure 4 shows a detailed breakdown of the distribution of prompt styles, which depicts the prevalence of each style and its relative frequency in structured data generation.

#### 3.1.2 Validation and accuracy assessment

Each generated output was validated against its expected dataset using a pattern-matching algorithm. This approach compared extracted attributes, identifying missing or incorrect fields. The results highlighted discrepancies in the generation process, particularly in verbosity and optional field inclusion.

By leveraging the 3 × 3 × 3 framework, Stage One established a robust basis for evaluating the performance of JSON, YAML, and Hybrid CSV/Prefix prompt styles. These datasets and their associated metrics were subsequently utilized in Stage Two (Section 3.2) to analyze accuracy, token cost, and generation time in greater depth.

#### 3.1.3 Personal stories dataset and validation

The *Personal Stories Dataset* evaluates GPT-4o's ability to generate narrative outputs that faithfully incorporate structured input attributes using the JSON, YAML, and the Hybrid CSV/Prefix prompt styles. This dataset simulates real-world scenarios where structured data is embedded seamlessly within natural language text while maintaining the integrity of all specified attributes. The generation process followed a systematic method to ensure consistency, accuracy, and validity.

**Figure 5:**
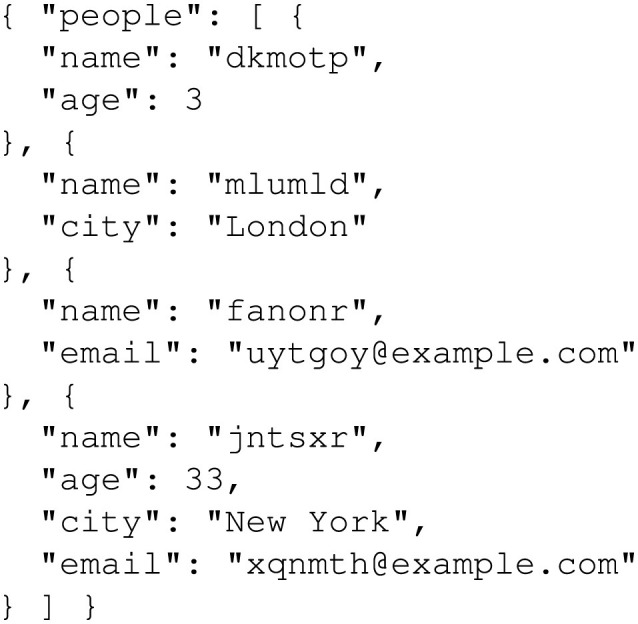
Random people generation example.

**Figure 6:**
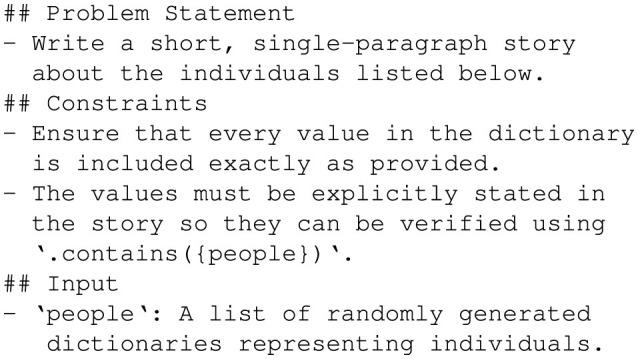
Prompt to generate personalized stories.

**Figure 7:**
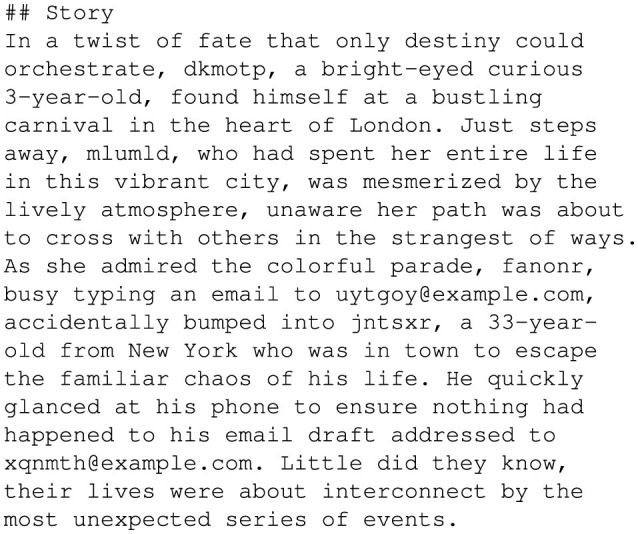
GPT-4o-generated personalized stories.

To create this dataset, random attributes were generated for fictional individuals, including fields like *name, age, city*, and optionally *email*, as shown in Figure 5. These attributes were structured in dictionaries where each entry represents a unique individual. The randomization ensured diversity and tested GPT-4o's capability to handle variations in input attributes effectively. Figure 6 shows the prompt used to guide GPT-4o in generating personalized stories. This prompt was designed to ensure each generated story explicitly included every attribute from the input dictionary in a short, coherent narrative. Moreover, this prompt enforced the inclusion of all attribute values exactly as provided, enabling rigorous validation of the generated outputs, as shown in Figure 7.

Validated stories were formatted into three distinct prompt styles for further analysis: (1) JSON for hierarchical structures with nested attributes, (2) YAML for human-readable formats, and (3) the Hybrid CSV/Prefix style that combined tabular headers with prefixed rows. These prompt styles provided diverse representations of the same data and were assessed for their accuracy, efficiency, and token usage. As discussed in Section 3.2, this analysis quantified the relative strengths and trade-offs of each prompt style in generating structured data using GPT-4o.

#### 3.1.4 Medical record dataset

The *Medical Record Dataset* evaluates GPT-4o's ability to generate structured medical records that accurately represent input attributes using the JSON, YAML, and the Hybrid CSV/Prefix prompt styles. This dataset simulates real-world scenarios where structured patient data is embedded within electronic medical records while preserving the completeness and correctness of all specified details. The generation process followed a systematic methodology to ensure consistency, accuracy, and validity.

To create the dataset, random attributes were generated for fictional individuals, including fields like *name*, and optionally *age, city*, and *email*. Medical-specific fields like *diagnosis, prescriptions*, and *doctor's notes* were excluded to focus the scope of this study. These attributes were organized into dictionaries, with each entry representing a unique individual. Figure 5 depicts random person generation for the *Medical Record Dataset*.

**Figure 8:**
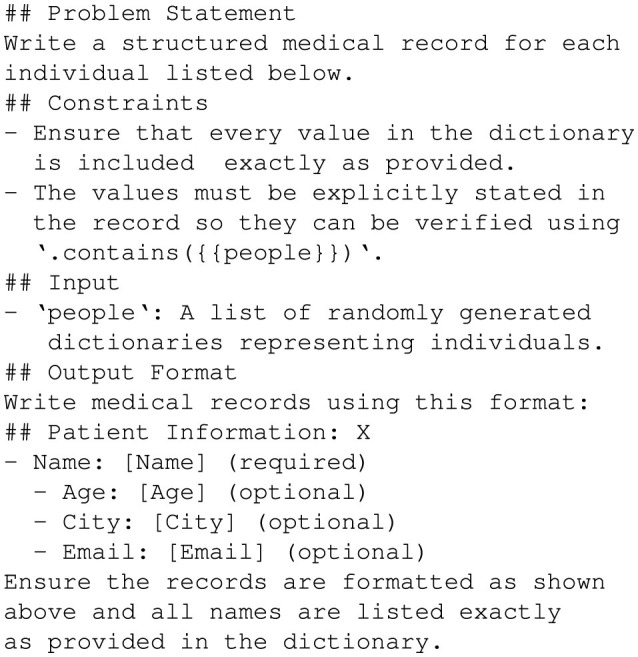
Prompt to generate medical records.

Randomizing attributes ensured diversity and tested each GPT-4o's ability to handle varying input fields effectively. The prompt shown in Figure 8

guided GPT-4o to generate structured medical records that explicitly include every attribute from the input dictionary. This prompt enforced the inclusion of all specified attributes and ensured the generated medical records followed the required structure and formatting.

The *Receipt Dataset* evaluates GPT-4o's ability to generate structured receipts that accurately represent input attributes using the JSON, YAML, and the Hybrid CSV/Prefix prompt styles. This dataset simulates real-world scenarios where structured transaction data is embedded within formal receipt templates, preserving the completeness and correctness of all specified details. The generation process followed a systematic method to ensure consistency, accuracy, and validity.

To create the dataset, random attributes were generated for fictional individuals, including fields like *name*, and optionally *age, city*, and *email*. These attributes were organized into dictionaries, with each entry representing a unique individual. Figure 5 depicts random person generation for the *Receipt Dataset*.

**Figure 9:**
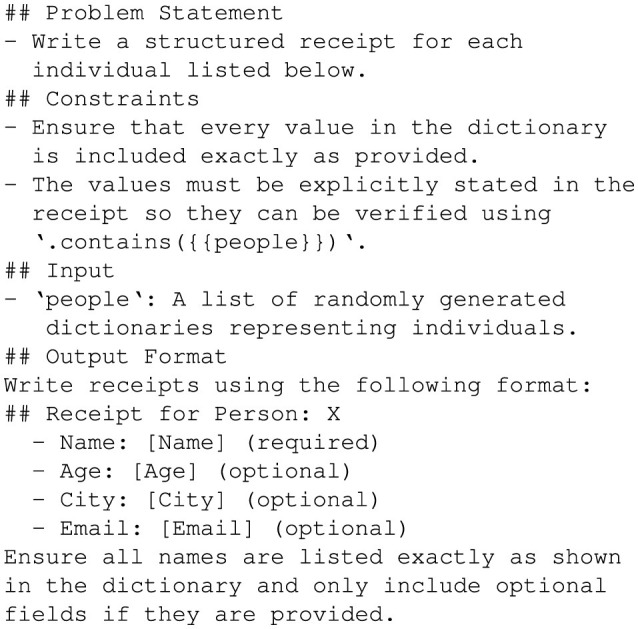
Prompt to generate receipt records.

Randomizing attributes ensured diversity and tested each GPT-4o's ability to handle varying input fields effectively. The prompt shown in Figure 9 guided GPT-4o to generate structured receipts that explicitly included every attribute from the input dictionary.

This prompt enforced the inclusion of all specified attributes and ensured the generated receipts follow the required structure and formatting.

### 3.2 Stage Two: assessment and refinement

The second stage of our study validated and assessed outputs generated by GPT-4o against the datasets described in Section 3.1. This stage measured the accuracy, efficiency, and token cost associated with all three prompt styles by building on the metrics identified during Stage One. Actionable insights were codified to evaluate the performance of each prompt style within the contexts of *Personal Stories, Medical Records*, and *Receipts*. Our analysis in Section 4 highlights the strengths, limitations, and trade-offs of these prompt styles in generating structured data.

Stage Two employed the JSON, YAML, and Hybrid CSV/Prefix distinct prompt styles to produce structured data in a standardized format. These prompt styles were tailored to represent the data effectively while testing GPT-4o's ability to generate outputs with accuracy, efficiency, and cost-effectiveness. Each style helped evaluate how well GPT-4o adhered to the provided structure and requirements, as described below.

**Figure 10:**
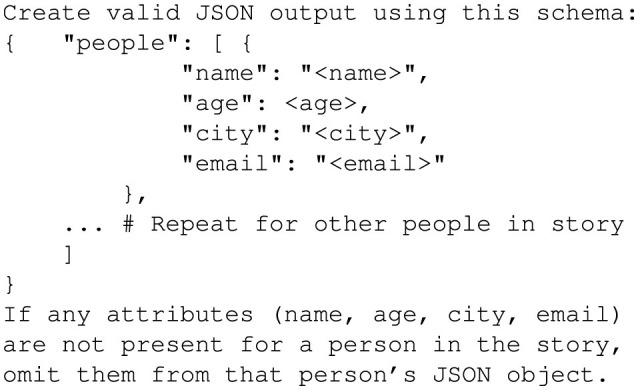
Prompt in JSON format.

The JSON prompt shown in Figure 10 instructs GPT-4o to create a structured JSON output adhering to a predefined schema.

This prompt style is hierarchical and suitable for applications requiring nested structures.

**Figure 11:**
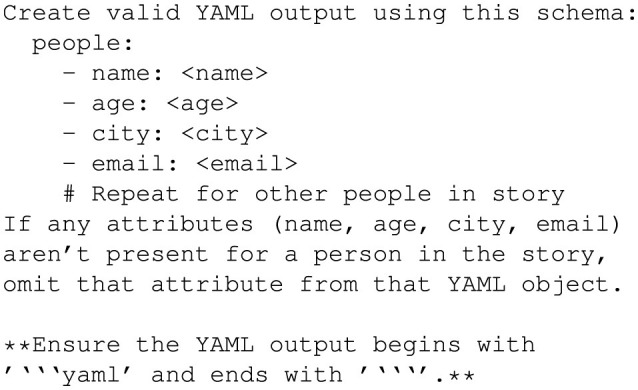
Prompt in YAML format.

**Figure 12:**
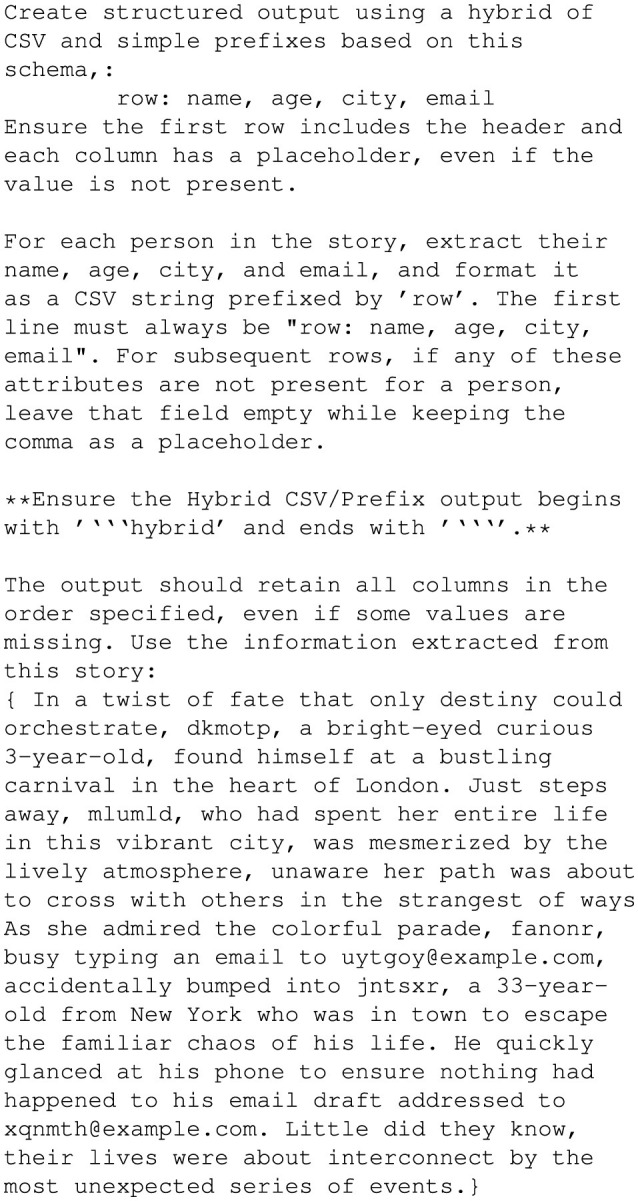
Prompt in the Hybrid CSV/Prefix format.

The YAML prompt in Figure 11 emphasizes human-readability while maintaining strict formatting standards, making it suitable for both human and machine interpretation. The Hybrid CSV/Prefix prompt in Figure 12 combines elements of tabular data and prefixed text, ensuring all attributes are included as placeholders even when their values are absent.

These three prompts were designed to evaluate how well GPT-4o interpreted and adhered to the provided instructions. The outputs generated from these prompts were validated against the *Personal Stories, Medical Records*, and *Receipts* datasets to measure accuracy. Using consistent prompts across all datasets ensured that the results accurately reflected GPT-4o's inherent capabilities without introducing bias from prompt-specific adjustments (Brown et al., 2020).

We evaluated the performance of the three prompt styles–JSON, YAML, and the Hybrid CSV/Prefix format–systematically by applying them to the three datasets: *Personal Stories, Medical Records*, and *Receipts*, exclusively using GPT-4o. Our assessment identified the strengths and weaknesses of each prompt style by comparing the outputs generated by GPT-4o against expected data using the following three measures:

- *Accuracy measures*, which calculated the percentage of attributes correctly included in generated outputs.
- *Token usage measures*, which evaluated the number of tokens consumed by each prompt style, as token efficiency directly impacts cost.
- *Time efficiency measures*, which computed response times for generating outputs to assess each prompt style's suitability for real-time or batch processing.

Our schema validation process (Arnes and Horsch, 2023) ensured every attribute from the input datasets was accurately reflected in the generated outputs. As discussed in Section 4, the findings from Stage Two provided actionable insights, enabling informed decisions on selecting the most effective prompt style for structured data generation with GPT-4o across various contexts.

## 4 Analysis of GPT-4o experiment results

This section provides a detailed analysis of GPT-4o's performance across the JSON, YAML, and the Hybrid CSV/Prefix prompt styles based on the assessment methodology outlined in Section 3.2. Our analysis focuses on the results of a single experiment conducted using the *Patient Information, Personal Story*, and *Receipt* datasets to evaluate GPT-4o across the accuracy, token usage, and time efficiency metrics.

### 4.1 Accuracy analysis for GPT-4o

**Figure 13:**
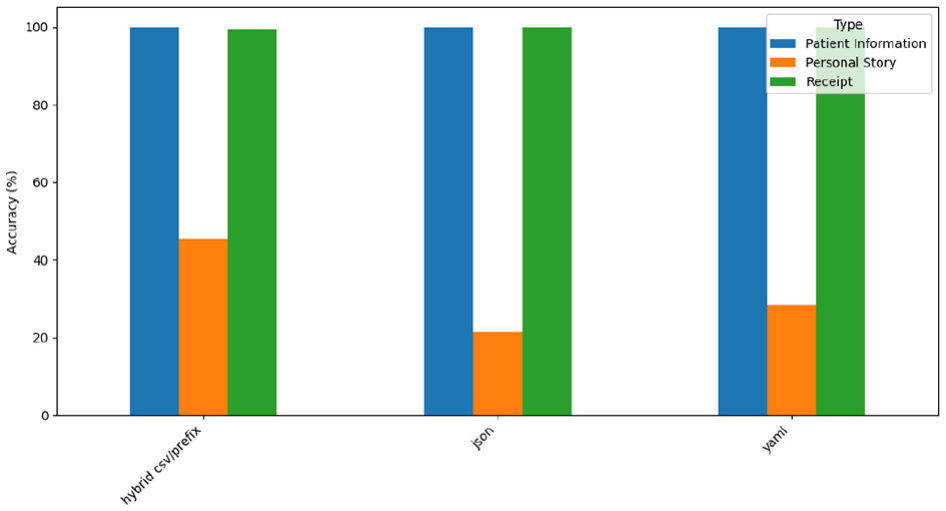
GPT-4o accuracy by prompt style and type.

Figure 13 depicts GPT-4o's accuracy across the three datasets.

GPT-4o achieves near-perfect accuracy for the *Patient Information* and *Receipt* structured datasets. However, it performs poorly for the narrative-style *Personal Story* dataset, where its accuracy drops to 20%–40%. This disparity reflects GPT-4o's strengths in processing structured formats while revealing challenges with unstructured, narrative outputs. JSON, YAML, and Hybrid CSV/Prefix deliver similarly high accuracy for structured data, though JSON exhibits variability with narrative data.

### 4.2 Token usage analysis for GPT-4o

**Figure 14:**
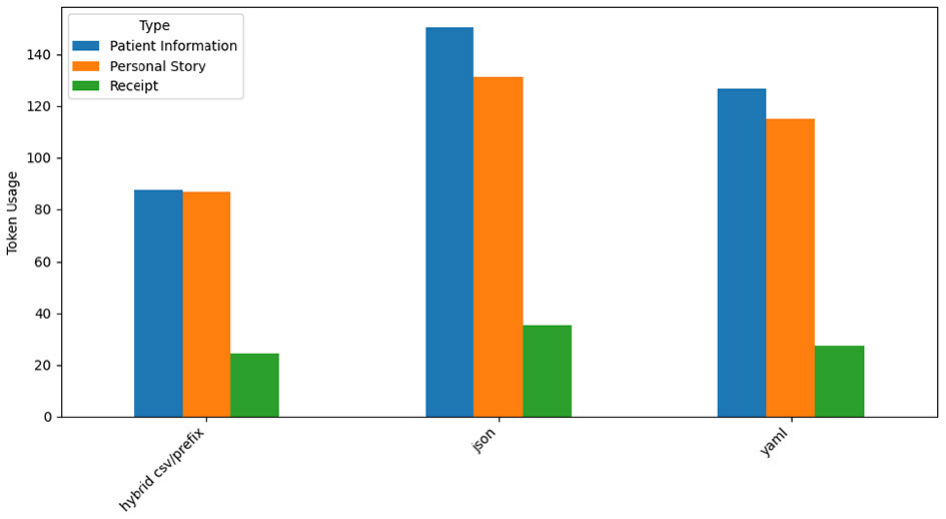
GPT-4o token usage by prompt style and type.

Figure 14 shows GPT-4o's token usage for the three prompt styles across datasets. Hybrid CSV/Prefix demonstrates the lowest token usage, making it the most efficient prompt style. JSON consumes the highest number of tokens due to its verbosity, particularly for hierarchical outputs in *Patient Information* and *Personal Story*. YAML exhibits moderate token efficiency, balancing conciseness and readability. These findings highlight Hybrid CSV/Prefix's suitability for cost-sensitive applications and JSON's trade-off between detailed representation and higher token costs.

### 4.3 Time analysis for GPT-4o

**Figure 15:**
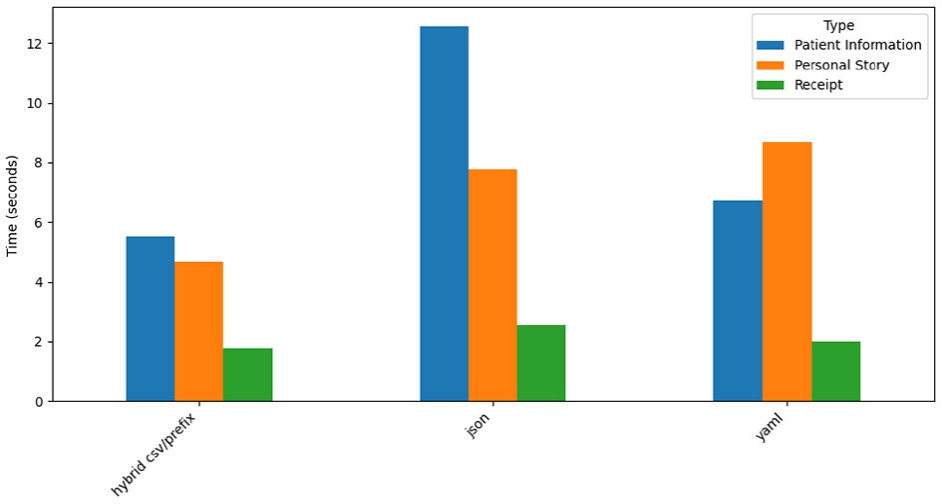
GPT-4o time taken by prompt style and type.

Figure 15 visualizes processing times for the three prompt styles across datasets. Hybrid CSV/Prefix achieves the fastest processing times, particularly for the simpler *Receipt* dataset, where generation times often fall below three seconds. JSON has the longest processing times, reflecting the complexity of generating hierarchical outputs, while YAML maintains moderate speeds. The *Patient Information* dataset consistently requires more time across all prompt styles due to its complex structure.

GPT-4o demonstrates strong performance for structured datasets (*Patient Information, Receipt*) but struggles with narrative-style data (*Personal Story*). Hybrid CSV/Prefix stands out as the most efficient prompt style, achieving the best balance of accuracy, token usage, and processing time. YAML offers a middle-ground solution, balancing readability and moderate efficiency, while JSON excels in hierarchical representation but incurs higher costs and processing times. These results underscore GPT-4o's strengths in structured data generation and highlight areas for improvement in narrative and unstructured contexts.

## 5 Comparative analysis of prompt styles averaged across datasets

This section compares the JSON, YAML, and Hybrid CSV/Prefix prompt styles averaged across the three *Personal Stories, Medical Records*, and *Receipts* datasets using the 3 × 3 × 3 framework and GPT-4o to evaluate their overall efficiency and effectiveness.

### 5.1 Accuracy comparison across prompt styles

**Figure 16:**
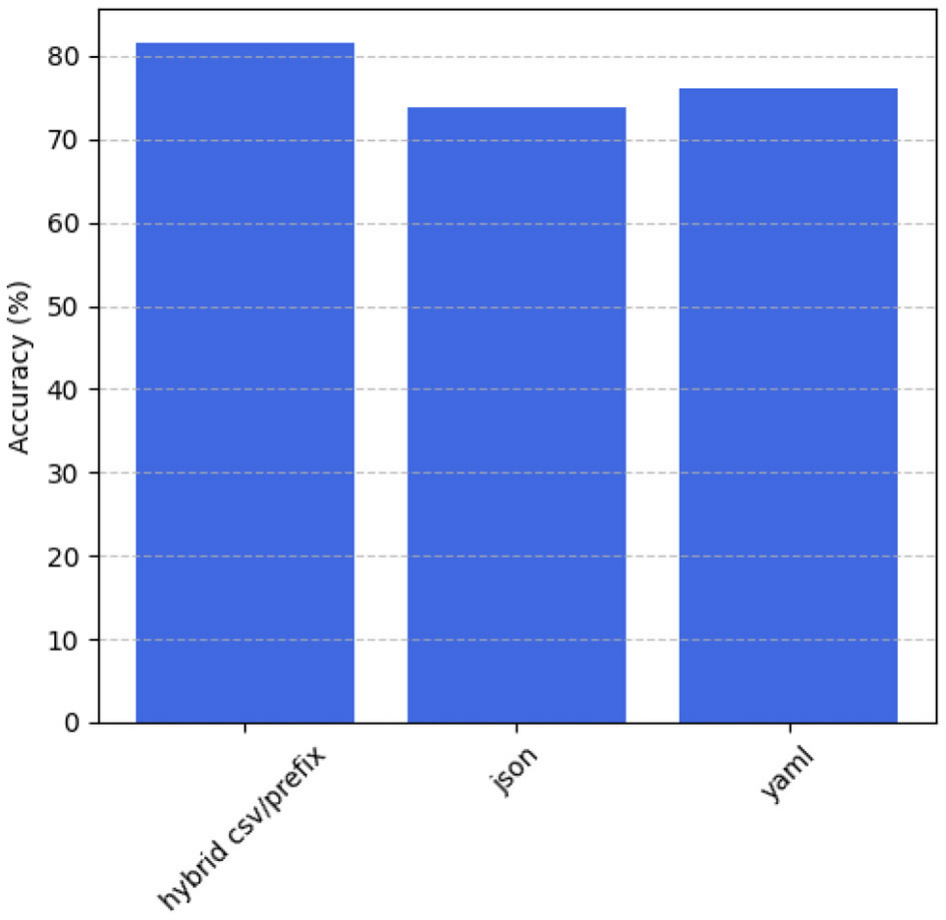
Comparative accuracy of prompt styles using GPT-4o.

Figure 16 visualizes the comparative accuracy performance of GPT-4o across various the Hybrid CSV/Prefix, JSON, and YAML prompt style. This figure shows how different structured prompting techniques affect the accuracy of generated outputs when evaluated across multiple datasets. The results indicate that Hybrid CSV/Prefix achieves the highest average accuracy at 82%, outperforming both YAML (76%) and JSON (74%), which suggests that Hybrid CSV/Prefix is the most effective technique for structured data generation since it consistently produces more accurate results.

YAML emerges as a balanced alternative, offering moderate accuracy with greater flexibility. Conversely, JSON, while commonly used for structured data representation, struggles with datasets requiring narrative complexity, leading to its comparatively lower accuracy. These findings highlight the impact of prompt structure on GPT-4o's ability to generate accurate structured data and emphasize that choosing the right prompt style significantly affects performance.

Figure 16 shows the average accuracy (%) achieved by GPT-4o when generating structured data using Hybrid CSV/Prefix, JSON, and YAML prompt styles. Hybrid CSV/Prefix achieves the highest accuracy, indicating that the structure of the prompt plays a significant role in output correctness.

We performed an ANOVA test to assess the accuracy differences across prompt styles statistically. The results yielded an F-statistic of 1.92 with a p-value of 0.088, indicating that while there are observable variations in accuracy, they are not statistically significant at the 5% level. Therefore, while Hybrid CSV/Prefix achieves the highest mean accuracy, the differences between prompt styles may not be large enough to reject the null hypothesis conclusively.

**Figure 17:**
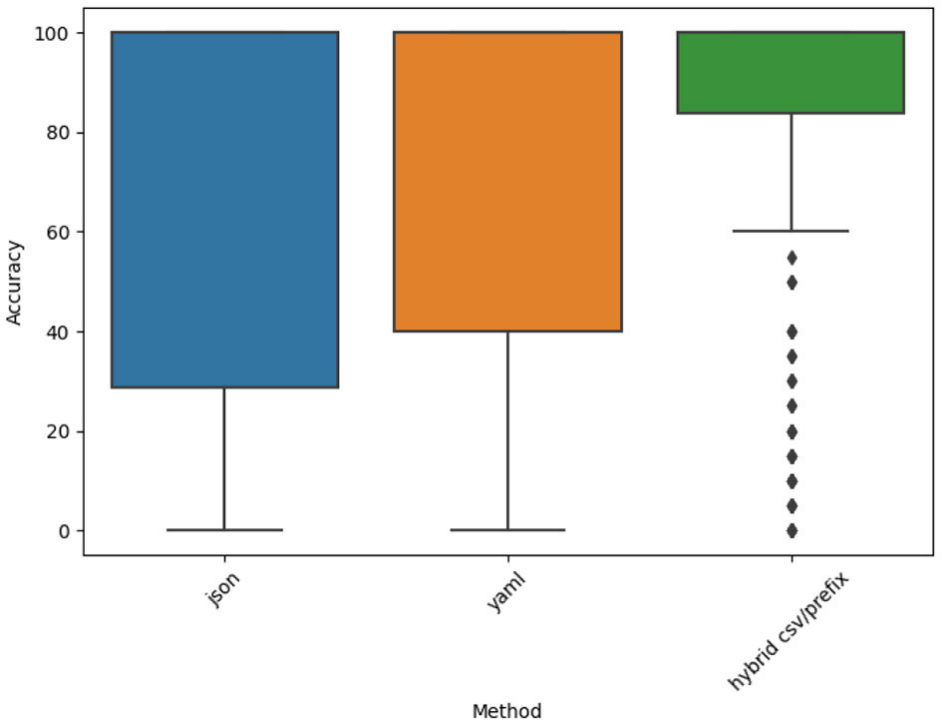
Accuracy distribution for the JSON, YAML, and Hybrid CSV/Prefix prompt styles.

To further investigate these variations, we conducted Tukey's HSD test to perform direct pairwise comparisons between prompt styles. Figure 17 presents the accuracy distribution across the JSON, YAML, and Hybrid CSV/Prefix prompt styles, showing the spread and consistency of accuracy values across datasets, i.e., JSON and YAML exhibit greater accuracy variability, with accuracy scores covering a wider range compared to Hybrid CSV/Prefix.

These results suggest that JSON and YAML sometimes produce highly accurate outputs but also inconsistent performance depending on the dataset. In contrast, Hybrid CSV/Prefix maintains a more stable accuracy distribution, with fewer extreme variations. These findings indicate that while Hybrid CSV/Prefix does not always outperform JSON and YAML in every instance, it provides more predictable and reliable performance across datasets.

**Figure 18:**
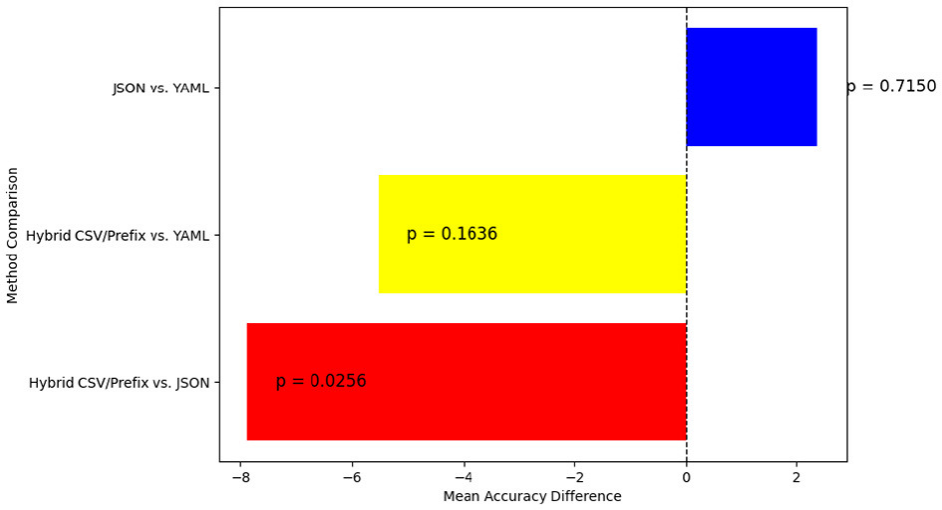
Pairwise accuracy differences between the JSON, YAML, and Hybrid CSV/Prefix prompt styles.

To gain deeper insights into the statistical significance of these differences, Tukey's HSD test was used for pairwise comparisons. Figure 18 highlights where significant accuracy variations exist. This figure depicts the statistical differences in accuracy using Tukey's HSD test. A significant difference (*p* < 0.05) is observed between Hybrid CSV/Prefix and JSON, while the comparisons between Hybrid CSV/Prefix vs. YAML and JSON vs. YAML do not reach statistical significance.

The following are key statistical findings from our experiments:

- **Hybrid CSV/Prefix vs. JSON**—JSON achieves a significantly higher accuracy than Hybrid CSV/Prefix, with a mean accuracy difference of 7.88 and a *p*-value of 0.0256. Since *p* < 0.05, this result confirms JSON's statistically significant advantage over Hybrid CSV/Prefix.
- **Hybrid CSV/Prefix vs. YAML**—YAML shows slightly higher accuracy than Hybrid CSV/Prefix, but the mean difference of 5.52 and *p*-value of 0.1636 indicate that this difference is not statistically significant.
- **JSON vs. YAML**—The mean accuracy difference is 2.37, with a *p*-value of 0.7150, confirming that JSON and YAML perform similarly in terms of accuracy.

These statistical findings reinforce that while JSON significantly outperforms Hybrid CSV/Prefix, YAML does not show a strong advantage over either method. This suggests that YAML remains a balanced alternative, while Hybrid CSV/Prefix, despite its structured nature, may require further refinements to improve accuracy.

### 5.2 Comparing the token usage of GPT-4o

**Figure 19:**
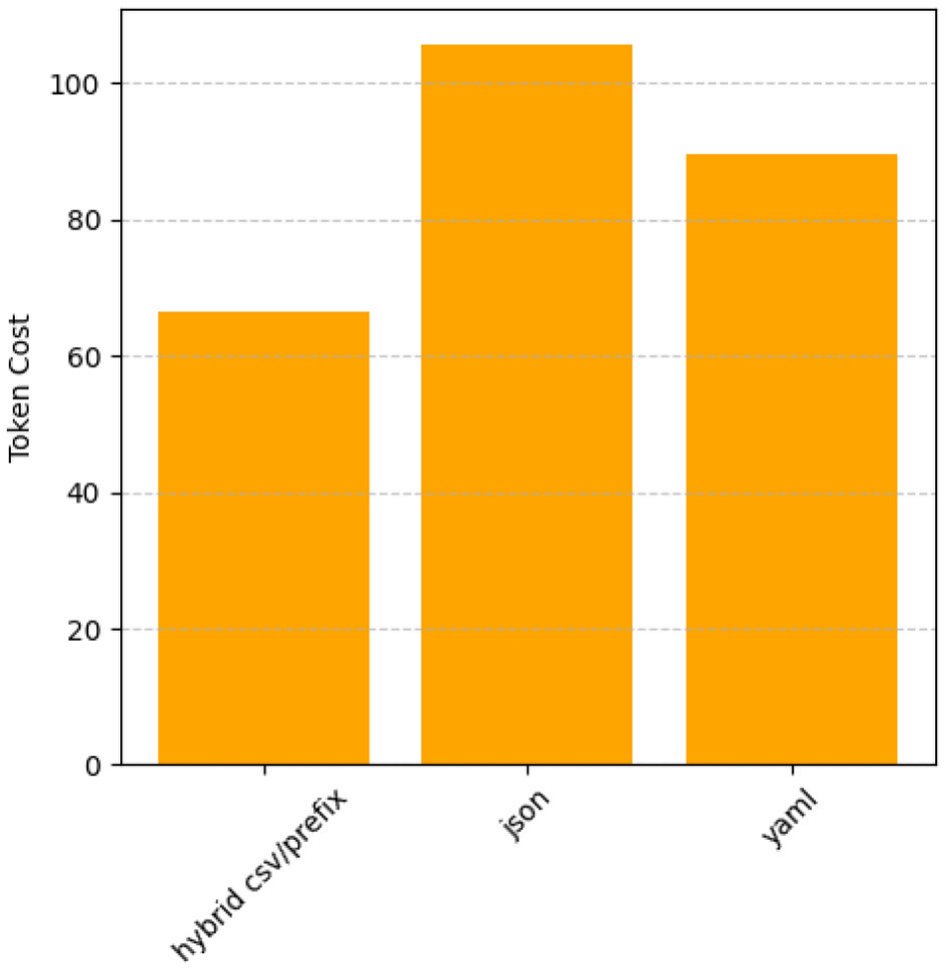
Token usage comparison by prompt style (averaged across datasets).

Figure 19 shows how Hybrid CSV/Prefix is the most token-efficient prompt style across datasets, making it ideal for cost-sensitive applications. Likewise, lYAML balances conciseness and detailed representation, offering moderate token usage. In contrast, JSON consumes the most tokens, especially for hierarchical or verbose outputs, indicating room for improvement in optimizing efficiency. These results underscore the suitability of Hybrid CSV/Prefix for tasks where minimizing resource consumption is critical.

### 5.3 Processing time comparison across prompt styles

**Figure 20:**
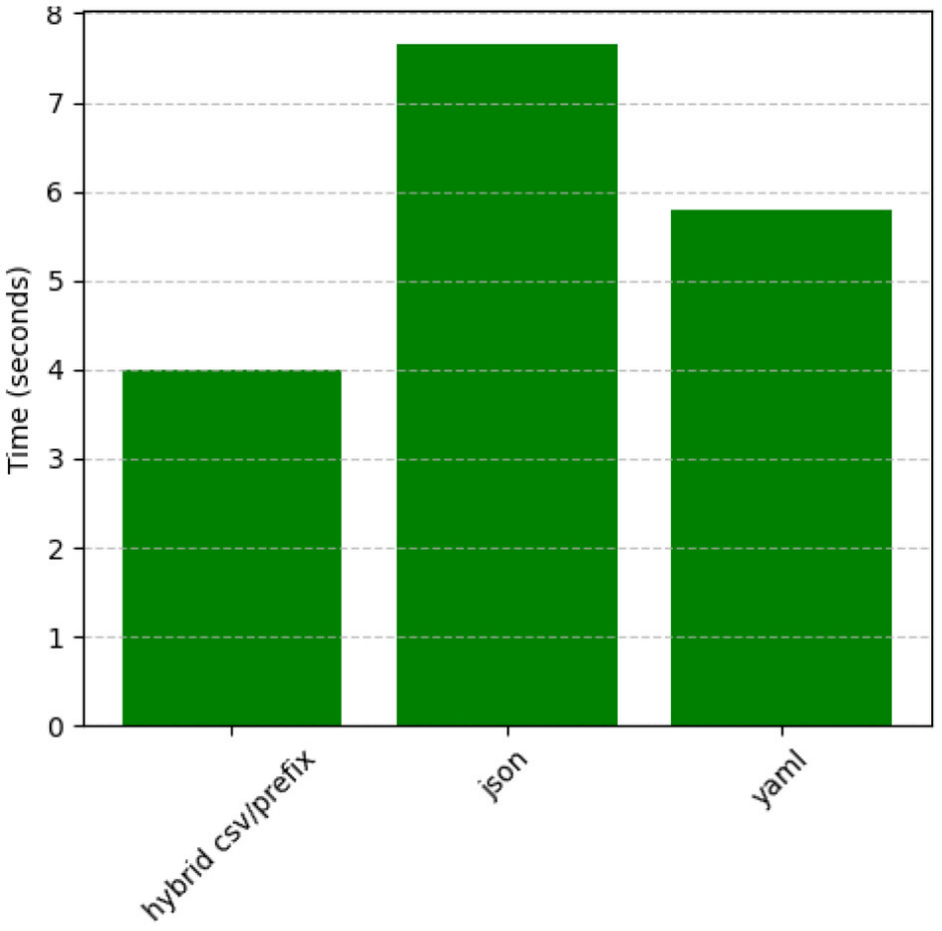
Processing time comparison by prompt style (averaged across datasets).

Figure 20 demonstrates that Hybrid CSV/Prefix also excels in processing time, consistently outperforming YAML and JSON. YAML maintains moderate speeds, balancing complexity and readability. JSON has the longest processing times, particularly for hierarchical datasets, further emphasizing the need for optimization. The efficiency of Hybrid CSV/Prefix makes it highly suitable for real-time and high-throughput applications.

Hybrid CSV/Prefix stands out as the most versatile and efficient prompt style, excelling in accuracy, token usage, and processing time. YAML provides a balanced alternative for scenarios requiring readability and moderate efficiency, while JSON remains specialized for hierarchical data but is less effective in cost and time-sensitive contexts. These insights offer practical guidance for selecting the optimal prompt style based on specific requirements and constraints.

## 6 Related work

**Figure 21:**
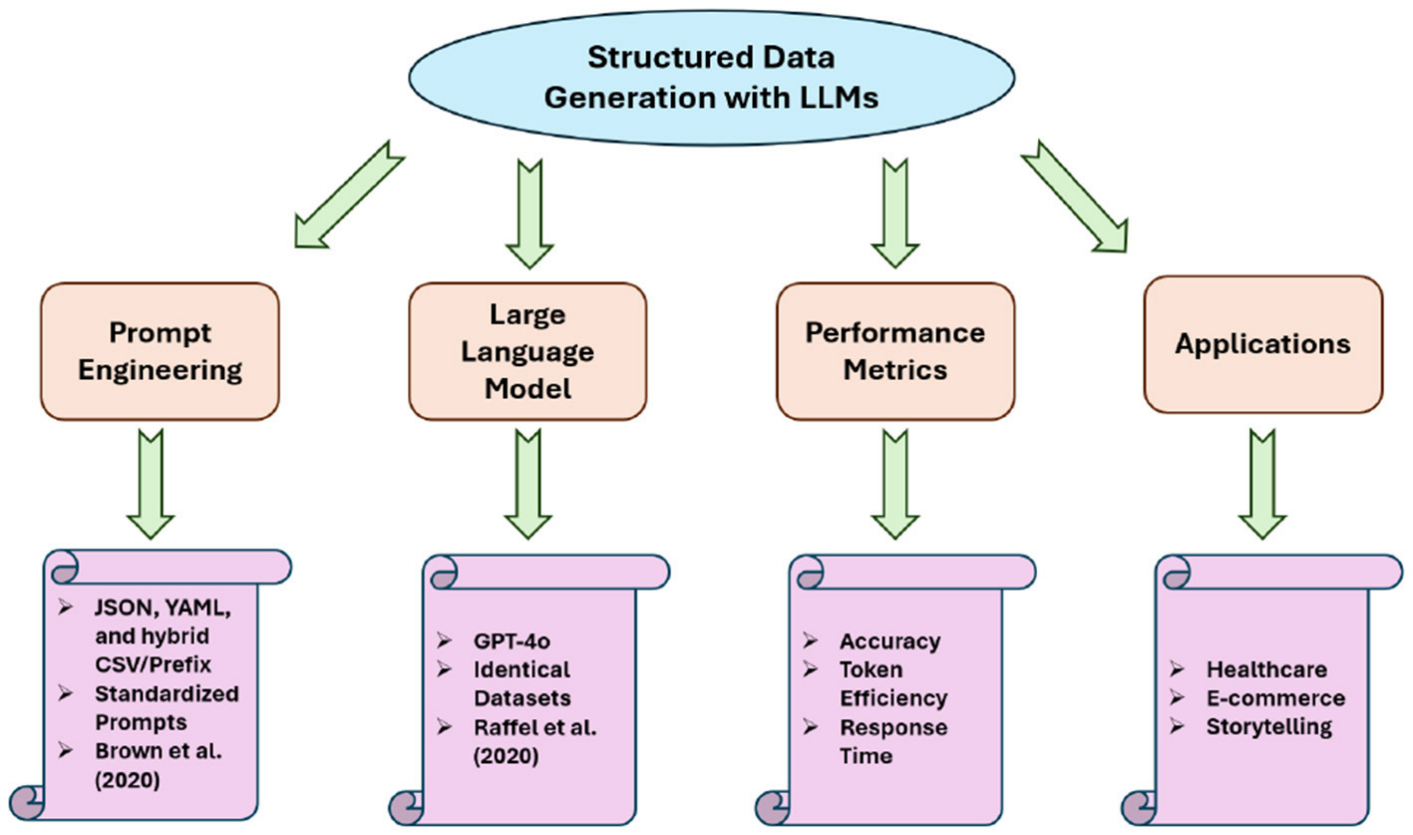
Related work research areas.

The study of LLMs has seen rapid advances in generating structured outputs for applications in diverse domains, including healthcare, e-commerce, and storytelling. Recent research has focused on prompt engineering techniques and performance evaluation metrics, especially for prompt styles like JSON, YAML, and hybrid CSV/Prefix. This section reviews relevant work in prompt design, performance benchmarking, and practical applications of structured data generation with LLMs, as shown in Figure 21.

### 6.1 Prompt engineering for structured outputs

Prompt engineering is central to optimize LLM performance for structured data generation. Prior work has explored the use of structured prompt styles, such as JSON, YAML, and a hybrid CSV/Prefix format, to guide model behavior effectively. Brown et al. (2020) demonstrated input formatting's importance for improving generative model outputs, highlighting JSON's flexibility for hierarchical structures and YAML's readability. Other studies investigated lightweight and hybrid approaches, such as combining tabular data with prefixed elements, to balance efficiency and accuracy. Unlike prior work that often tailors prompts for specific LLMs, our study uses a consistent set of prompts across datasets and prompt styles, ensuring fair evaluation of GPT-4o's capabilities with standardized input styles.

### 6.2 Performance metrics: measuring efficiency, accuracy, and cost-effectiveness

Metrics like accuracy, token efficiency, and response time are critical for assessing LLMs in generating structured outputs. Vaswani et al. (2017) and subsequent studies emphasized the importance of token usage as a measure of scalability and cost-effectiveness. Recent work by OpenAI (Achiam et al., 2023) explored accuracy in generating structured outputs for use cases like medical records and financial summaries, emphasizing the trade-offs between verbose outputs and efficiency. Our study builds on this foundation by providing a comprehensive analysis of GPT-4o's performance across the Hybrid CSV/Prefix, JSON, and YAML prompt styles, benchmarking its strengths and limitations in structured and semi-structured contexts.

### 6.3 Applications of structured data generation with GPT-4o

GPT-4o has shown significant potential in domains requiring structured data, including healthcare, e-commerce, and storytelling. For instance, Kumichev et al. (2024) demonstrated the generation of synthetic medical records for data augmentation, while other studies leveraged LLMs for generating product descriptions and receipt summaries (Kedia et al., 2021). These use cases align with our *Personal Stories, Medical Records*, and *Receipts* datasets, enabling realistic evaluation of GPT-4o's ability to generate structured outputs in practical applications. Our work emphasizes GPT-4o's adaptability to diverse prompt styles, including the efficiency of YAML for human-readable outputs, JSON for hierarchical data, and Hybrid CSV/Prefix for balanced versatility.

### 6.4 Advantages and trade-offs for structured prompt styles

The JSON, YAML, and Hybrid CSV/Prefix prompt styles each offer advantages for structured data generation, as highlighted in prior research. JSON is commonly used for hierarchical and nested data, and is thus well-suited for tasks requiring precise structure (Brown et al., 2020). YAML is known for its human-readable design, which makes it particularly effective when interpretability is critical (Eriksson and Hallberg, 2011). Hybrid CSV/Prefix is a more recent approach that combines the simplicity of tabular formats with structured elements, achieving a balance between token efficiency and adaptability (Ball et al., 2007).

### 6.5 Structured data validation and schema enforcement

Prior studies have highlighted the importance of schema validation for ensuring data consistency and correctness in structured outputs. Du et al. (2020) introduced schema-guided generation techniques to validate and enforce structural constraints, particularly for JSON and YAML outputs. Schema-aware approaches were shown to improve accuracy by reducing parsing errors and increasing adherence to predefined structures.

Our study builds on these insights by evaluating GPT-4o's performance across prompt styles to reach the following conclusions, which are consistent with prior work:

- JSON demonstrated strengths in handling complex tasks but exhibited higher token costs (Liu et al., 2023).
- YAML offered moderate accuracy and efficiency, aligning with its emphasis on clarity (Achiam et al., 2023).
- Hybrid CSV/Prefix excelled in efficiency and adaptability, confirming its potential for token-sensitive applications (Guo, 2023).

Our evaluation in this paper highlights GPT-4o's ability to balance accuracy, token usage, and processing time across these prompt styles. We also identify opportunities for further optimization, particularly in handling hierarchical and narrative data contexts.

## 7 Concluding remarks

This paper presented a detailed methodology, comprehensive analysis, and practical recommendations based on our evaluation of applying three prompt styles–Hybrid CSV/Prefix, JSON, and YAML–to structured data generation tasks with GPT-4o. Our study highlighted key trade-offs and insights into the use of these prompt styles for diverse applications. The following are lessons learned from the research conducted in this paper:

- **Prompt selection depends on context and trade-offs**. Each prompt style offers unique advantages depending on the application. For instance, YAML provides excellent human-readability and is well-suited for semi-structured data, such as receipts or user-facing contexts. JSON excels in hierarchical data representation, making it valuable for complex tasks like medical records, though it incurs higher token costs. The Hybrid CSV/Prefix prompt style balances efficiency and accuracy, making it a versatile choice for transactional or tabular datasets.
- **Hybrid CSV/Prefix balances accuracy and efficiency**. Among the prompt styles tested, Hybrid CSV/Prefix (Ball et al., 2007) demonstrated consistent high performance across structured and semi-structured tasks, achieving notable accuracy while minimizing token usage and processing time. This prompt style's adaptability highlights its value for domains prioritizing cost-effectiveness and operational speed.
- **YAML emphasizes human-readability**, which ensures usability in scenarios (Eriksson and Hallberg, 2011) where interpretability is a key requirement. It performed well in tasks requiring clarity and structure but showed moderate token efficiency compared to Hybrid CSV/Prefix.
- **JSON excels in hierarchical representation** and is the most effective prompt style for tasks requiring detailed hierarchical outputs. However, its verbosity contributes to higher token usage and longer processing times, indicating the need for optimization in resource-constrained environments.
- **Prompt design impacts accuracy and efficiency**. The findings underscore the importance of selecting a prompt style that aligns with the dataset's complexity and application requirements. For structured tasks, careful design of prompts–such as using YAML for clarity, Hybrid CSV/Prefix for versatility, or JSON for hierarchical data–can significantly improve both accuracy and efficiency.
- **Efficiency gains with GPT-4o**. GPT-4o demonstrated strong performance across all three prompt styles, excelling in token efficiency and time across structured and semi-structured datasets. These results emphasize its adaptability for cost-sensitive applications while maintaining reliable accuracy.
- **There's a “double-edged sword” with LLMs**. While prompting enables significant gains in accuracy, efficiency, and cost-effectiveness for structured data generation, it also introduces challenges. For example, verbose styles like JSON can yield higher token costs and slower processing times. Likewise, limitations in narrative accuracy raise concerns about using AI-generated outputs in safety-critical domains. These challenges motivate balanced approaches to prompt design that maximize benefits while mitigating risks.

By fostering responsible and effective use of prompting, our study contributes to future work on the responsible application of AI in diverse domains. For example, iterative refinement holds significant potential to improve the quality of LLM outputs. Likewise, we are exploring dynamic prompt optimization techniques and their impact on structured data generation across multiple LLMs. We are also expanding our scope to other structured data prompt styles, such as CSV, API function calls, or prefix-based styles, to provide a more comprehensive understanding of how these styles influence performance in diverse application domains.

## Data Availability

The datasets presented in this study can be found in online repositories. The names of the repository/repositories and accession number(s) can be found below: https://github.com/elnashara/EfficientStructuringMethods/tree/main/data.
